# Complete mitochondrial genome of *Eupteryx*(*Stacla*) *minusula* (Hemiptera: Cicadellidae: Typhlocybinae) from China

**DOI:** 10.1080/23802359.2020.1775146

**Published:** 2020-06-08

**Authors:** Xiao Yang, Zhouwei Yuan, Can Li, Yuehua Song

**Affiliations:** a School of Karst Science, Guizhou Normal University/State Engineering Technology Institute for Karst Desertification Control, Guiyang, China; b Guizhou Provincial Key Laboratory for Rare Animal and Economic Insect of the Mountainous Region, Guiyang University, Guiyang, China

**Keywords:** *Eupteryx (Stacla) minusula*, leafhopper, mitochondrial genome

## Abstract

The complete mitochondrial genome of the leafhopper species *Eupteryx* (*Stacla*) *minusula* (Hemiptera: Cicadellidae: Typhlocybinae) are sequenced and annotated. The mitochondrial genome is 16945 bp, and nucleotide composition of the whole mitogenome is highly A + T biased (A: 43.6%; T: 35.2%, G: 11.3%, C: 9.9%). 11 PCGs have ATN as the start codon, except for *atp8* and *nad5* genes have TTG. The conventional termination codons (TAA or TAG) occur in 11 PCGs, while *cox2* and *nad5* uses incomplete codon (T) as termination codon. The complete mitogenome sequence of *Eupteryx* (*Stacla*) *minusula* is available in the GenBank with accession number: MN910279.

The leafhopper genus *Eupteryx* belongs to the tribe Typhlocybini and distributes in the Palearctic, Nearctic, Oriental and Ethiopian regions (Dworakowska 1979). The genus *Eupteryx* was established by Curtis (1829) with *Cicada atropunctaca* Goeze as its type species and includes two subgenera *Eupteryx* and *Stacla*. And the *Eupteryx* (*Stacla*) *minusula* was reported in 1929 (Lindberg 1929). Until now, there are eleven known species of the subgenus *Stacla*, all restricted to the Oriental Region (Hou et al. 2016). In Europe and North America, some species of *Eupteryx* are important pests of medical and culinary herbs in terms of feeding damage (Henke et al. 2013). This genus now contains 120 species, all species are very similar in coloration and difficult to distinguish externally, but the structure of male genitalia are markedly different. A male adult of *E.* (*Stacla*) *minusula* was selected as specimen. All examined samples were collected from Huajiang, Guizhou Province, China. Thetotal DNA was extracted from entire body without abdomen. Phylogenetic analysis was carried out on the basis of 15 available mitogenomes of Hemipteran insects in GenBank including newly sequenced mitogenome of *E.* (*Stacla*) *minusula* with Neighbor-joining (NJ) methods using MEGA ver 6.0. The whole body was preserved in ethanol and deposited in the insect specimen room of Guizhou Normal University with an accession number GZNU-ELS-2019011.

The complete mitochondrial genome of *E.* (*Stacla*) *minusula* is 16,945 bp in size (GenBank accession number: MN910279), contains the set of 37 typical mitochondrial genes (13 protein-coding genes, 22 tRNA genes, and 2 rRNA genes), and an AT-rich region. The nucleotide composition of the whole mitogenome is highly A + T biased(A: 43.6%; T: 35.2%, G: 11.3%, C: 9.9%). All 13 PCGs started with the canonical putative start codon ATN except for the *nad5* and *atp8* which started with TTG instead. 11 genes shared complete termination codon TAA/TAG, while *cox2* and *nada* used incomplete stop codon (a single T). All tRNA genes are identified by ARWEN version 1.2 software (Laslett and Canback 2008). The 16S rRNA gene is 1161 bp long and located between *tRNL2* and *tRNV*; the 12S rRNA gene is 734 bp length and located after *tRNV*.

The nucleotides sequences of 13 PCGs of *E.* (*Stacla*) *minusula* and other 13 leafhopper species in family Cicadellidae were used to construct phylogenetic tree (Figure 1). The results showed that *E.* (*Stacla*) *minusula* and other two species of subfamily Typhlocybinae were clustered into one clade, which was separated from other subfamilies. Therefore, the result obtained in this study provided new molecular data for the evolution of the subfamily Typhlocybinae.

**Figure 1.**
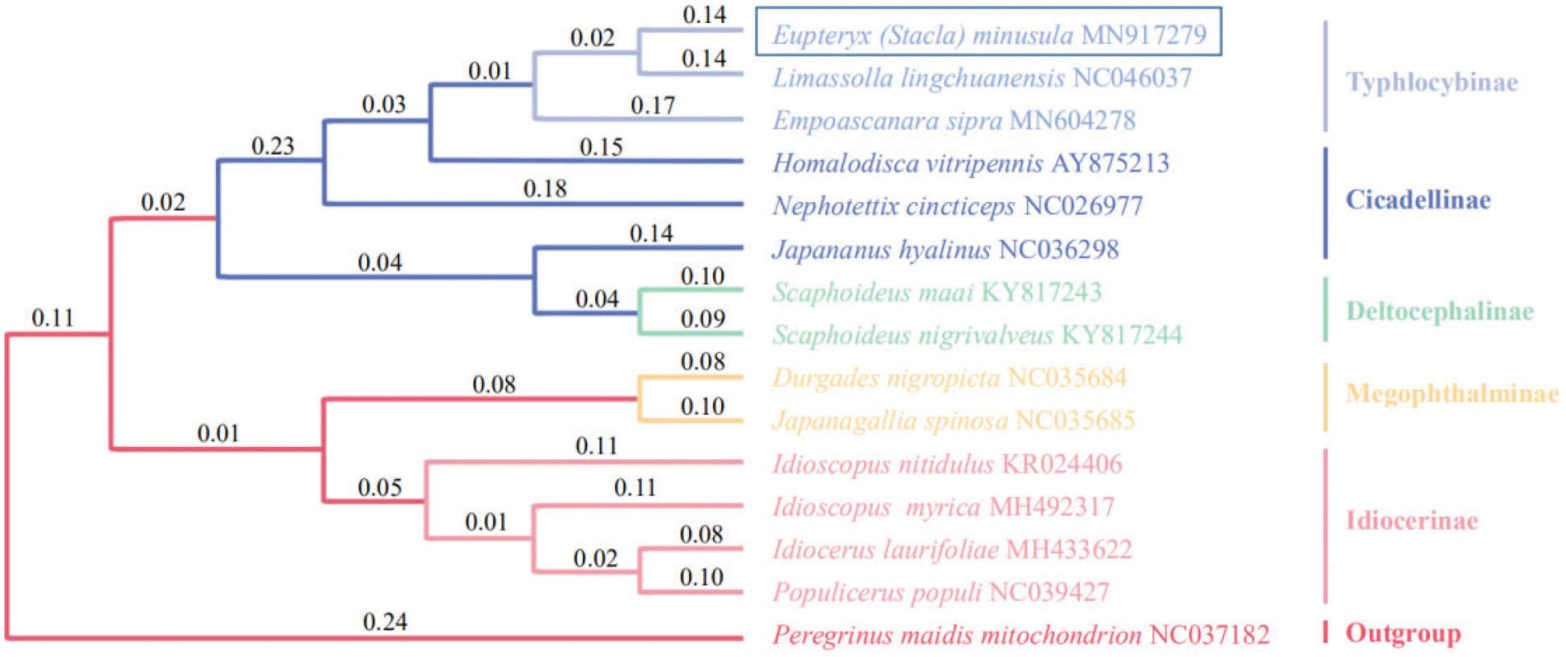
Phylogenetic tree showing the relationship between *E. (Stacla) minusula* and 13 other leafhoppers in inner group based on neighbour-joining method.

## Data Availability

The authors confirm that the data supporting the results of this study can be obtained from the corresponding author, upon reasonable request, or openly available in NCBI GenBank database at (https://www.ncbi.nlm.nih.gov/) with the accession number is MN917279.
